# Case Report: Successful management of psychogenic non-epileptic seizures with intranasal esketamine

**DOI:** 10.3389/fpsyt.2025.1527166

**Published:** 2025-04-22

**Authors:** Muneeb Hashmi, Padmasini Mugunthan, Priyal Jain, Tharun Nimmakayala, Sarah Alnaher

**Affiliations:** ^1^ College of Medicine, Texas A&M University College of Medicine, Bryan, TX, United States; ^2^ Medical Institute, Tambov State Medical University, Tambov, Russia; ^3^ Grant Government Medical College, Mumbai, India; ^4^ Apollo Institute of Medical Sciences and Research, Chittoor, India; ^5^ College of Medicine, Gulf Medical University, Ajman, United Arab Emirates

**Keywords:** psychogenic non-epileptic seizures, treatment-resistant depression, esketamine, functional seizures, post-traumatic stress disorder, psychotrauma, neuropsychiatric disorders, anxiety

## Abstract

**Introduction:**

Psychogenic non-epileptic seizures (PNES), or functional seizures (FS), are episodes that resemble epileptic seizures but may be psychological in origin. Unlike epileptic seizures, which are linked to abnormal electrical activity in the brain, functional seizures may be associated with psychological and/or physical distress, and do not show the same electrical patterns on an electroencephalogram (EEG). Esketamine, a derivative of the anesthetic ketamine, is approved by the U.S. Food and Drug Administration (FDA) for treatment-resistant depression (TRD) and major depressive disorder (MDD) with suicidal thoughts or actions.

**Methods/Results:**

This report discusses a patient with TRD and PNES, where the administration of esketamine effectively resolved both conditions.

**Discussion:**

It explores the potential therapeutic effects of esketamine on PNES, in addition to its antidepressant properties.

## Introduction

An epileptic seizure is a sudden disruption in the electrical activity of the brain that causes rapid, involuntary movements, abnormal behaviors, and/or loss of consciousness. Psychogenic non-epileptic seizures (PNES), or functional seizures, are associated with similar symptoms but instead may result from psychological and/or physical distress (1, 2). Electroencephalogram (EEG) monitoring of PNES patients confirms the lack of irregular bursts of electrical activity in the brain during episodes, and is the primary diagnostic test used to differentiate PNES from epileptic seizures. The fifth edition of the Diagnostic and Statistical Manual of Mental Disorders (DSM-5) identifies PNES as a symptom of conversion or functional neurological symptom disorder, and states that anxiety and depressive disorders are the most common comorbidities (3). The International Classification of Diseases 11th Revision (ICD-11) classifies PNES as a dissociative neurological symptom disorder. A study estimates the prevalence of PNES to be 2-33/100,000 per year, while the incidence is 1.4-5/100,000 per year (4). Current treatment options consist of psychotherapy such as cognitive behavioral therapy (CBT) (5), and, if necessary, medication to treat the underlying condition. Antiepileptic medication is not effective in treating PNES (6).

Ketamine is a dissociative anesthetic known for its antidepressant properties (7), although its racemic form is not approved by the U.S. Food and Drug Administration (FDA) for psychiatric treatment (8). The S-enantiomer of ketamine, known as esketamine, received FDA approval in 2019 for the treatment of treatment-resistant depression (TRD), which is characterized by a lack of response to two or more antidepressants after at least six weeks of treatment. Esketamine was subsequently also approved to treat major depressive disorder (MDD) with acute suicidal ideations or behaviors (8). This case report discusses a patient with TRD and PNES who was treated with intranasal esketamine. The treatment successfully alleviated depressive symptoms and resolved PNES.

## Case presentation

**Figure 1:**
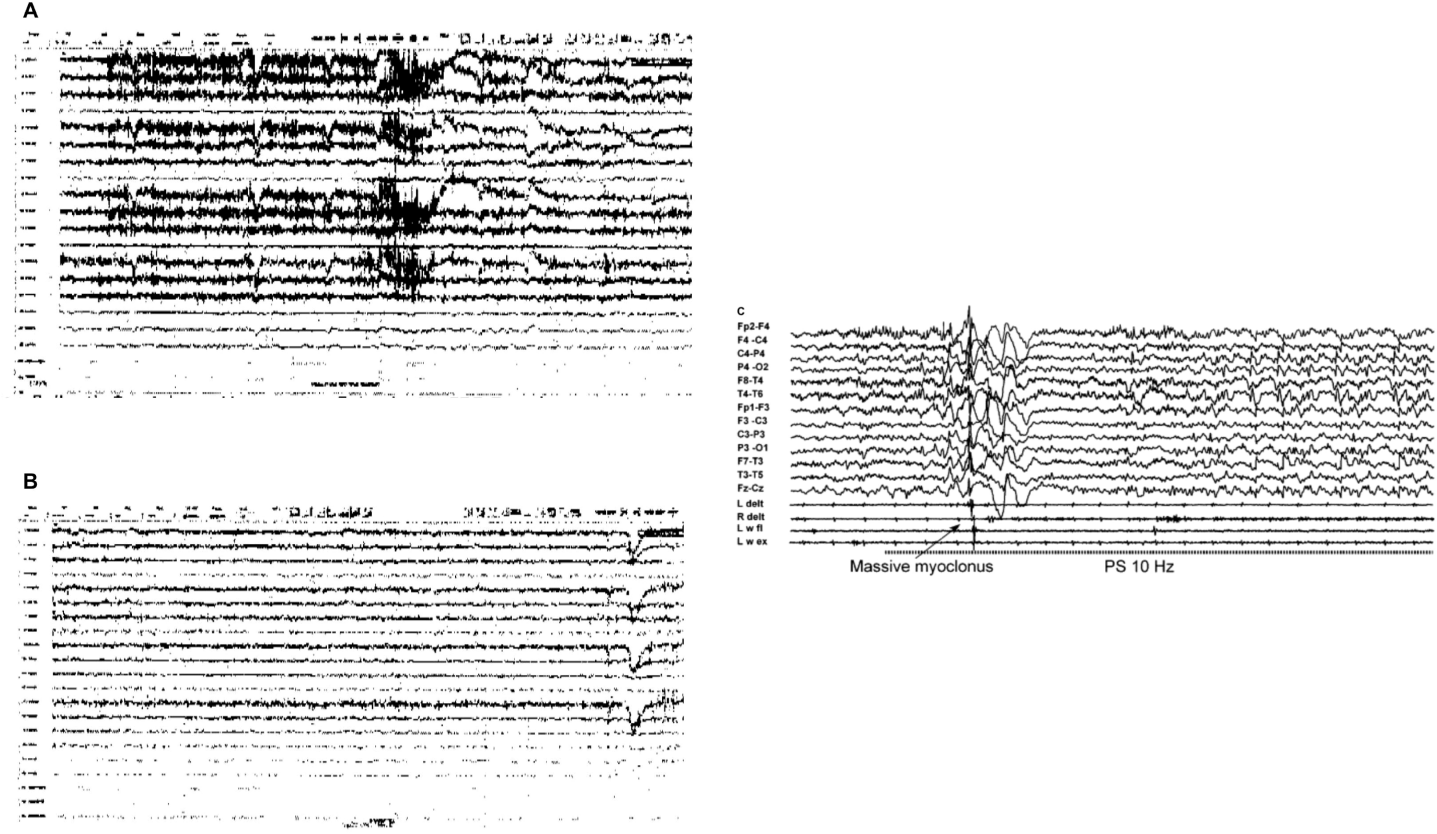
Patient’s Electroencephalogram (EEG) Images **(A, B)** Indicative of Psychogenic Non-Epileptic Seizures (PNES) and Example of Epileptic Seizure Activity **(C)**. **(A)** Non-epileptic paroxysmal event (seizure with no EEG correlate); **(B)** Psychogenic non-epileptic semiology: hypermotor episode with no EEG correlate. Both events during this continuous video EEG study were not associated with epileptiform abnormalities and thus diagnostic of PNES. **(C)** Example of EEG scan during epileptic seizure (21).

The patient is a 45-year-old married Caucasian female. She was first diagnosed with depression at the age of 18, when she was treated by her primary care physician. Over subsequent years, she was treated with antidepressants including escitalopram, trazodone, and fluoxetine. Her mood remained stable over the years. Recently, she started experiencing episodes of generalized tonic-clonic seizures, which did not respond to multiple antiepileptic medications. Her neurologist performed a sleep deprived EEG and, after confirming a lack of abnormal electrical activity during a seizure episode, diagnosed her with PNES (see **Figure 1**). In addition to the seizure episodes, she suffered an episode of speech deficits which rapidly resolved. She also suffered loss of peripheral vision and was diagnosed with optic neuropathy. A magnetic resonance imaging (MRI) scan of her brain was within normal limits. The burden of her seizures caused recurrence of severe depression, with complaints of poor sleep, decreased interest, decreased energy, poor concentrating ability, decreased appetite with significant weight loss (from 260 to 191 lbs), psychomotor retardation, and crying spells. She felt hopeless in that she was a burden on her family because she had not been able to function. Her primary care physician prescribed a medication regimen of fluoxetine and alprazolam with no significant benefit.

A few months later, she was admitted to an inpatient psychiatric facility for suicidal ideations and stabilized on fluoxetine 60 mg daily, trazodone 150 mg at night, and hydroxyzine 50 mg twice daily as needed. After discharge from hospital, she continued to experience seizure episodes. She was readmitted to a psychiatric hospital following a suicide attempt by overdose, and was later discharged on a medication regimen of fluoxetine 80 mg daily, quetiapine 100 mg at night, and clonazepam 0.25 mg twice daily. The fluoxetine dosage was increased to 80 mg at the patient’s request, as she had previously been unresponsive to tricyclic antidepressants and considered electroconvulsive therapy (ECT) too invasive. Despite these changes, she reported worsening depression and frequent generalized tonic-clonic seizures. She was subsequently evaluated by an outpatient psychiatrist, to whom the patient disclosed a history of severe emotional and physical abuse by her ex-husband. She reported ongoing trauma-related symptoms, including avoidance behaviors, heightened awareness, startle response, and nightmares. She was given an additional diagnosis of post-traumatic stress disorder (PTSD), and referred for regular therapy sessions, which have been integral in managing these symptoms. Bupropion 300 mg daily was also added to her existing medication regimen.

Due to a history of a poor response to more than two antidepressants, she met criteria for a diagnosis of treatment-resistant depression (TRD) and for treatment with esketamine (Spravato). She was prescribed 56 mg intranasally twice a week for 4 weeks, followed by maintenance therapy with 84 mg once a week. Cariprazine 1.5 mg daily was also added to augment fluoxetine; the rest of her regimen remained the same. Since initiating esketamine, she has not experienced functional seizures and has shown consistent improvement in depressive symptoms. Her Hamilton Depression Rating Scale (HAM-D) score decreased from a pre-treatment score of 23 to a score of 7. She has not reported side effects of esketamine.

She has returned to work and is now functional, reporting a much happier state of mind, especially since she has not had any PNES episodes since starting esketamine. Beyond returning to work, she has noted a significant improvement in her overall daily functioning and quality of life.

## Discussion

Our report explores the unexpected impact of esketamine, a medication for treatment-resistant depression (TRD), on a patient with psychogenic non-epileptic seizures (PNES). By examining the psychological and physiological factors contributing to PNES, along with esketamine’s effects and clinical effectiveness, we aim to uncover additional therapeutic benefits beyond reducing depressive symptoms. The resolution of PNES in our patient following esketamine treatment raises important questions about its potential to treat both TRD and PNES, prompting further investigation into its dual efficacy.

Treatment-resistant depression (TRD) refers to major depressive disorder (MDD) that does not respond to at least two different antidepressant regimens at adequate dose and duration (9). Individuals with TRD often present with a constellation of symptoms, including both psychological and physical manifestations. In addition to core symptoms such as depressed mood and anhedonia, severe physical or psychosomatic symptoms – tremors, seizures, and autonomic dysfunction – are potential presentations of MDD/TRD (10). The interconnectedness of physical and depressive symptoms is crucial to recognize, especially in this case of comorbid TRD and PNES.

PNES are believed to arise from a complex interplay of psychological and physiological factors. Baslet describes PNES as a psycho-neurologic illness, often manifesting as a physical response to psychological distress (11). This condition is frequently associated with a high prevalence of comorbid psychiatric conditions, such as mood disorders, anxiety disorders, PTSD, and dissociative disorders (3). The biopsychosocial model suggests that a combination of biological, psychological, and social factors contributes to the onset and persistence of PNES (12).

Trauma and psychological distress play a significant role in the genesis of PNES. High rates of abuse and trauma are reported among individuals with PNES, with studies indicating that lifetime rates of physical and/or sexual abuse in these individuals range from 50% to 77% (11, 13, 14). The development and perpetuation of PNES are influenced by a complex interaction of neurological conditions, new illnesses, life events, relationship conflicts, and cognitive and behavioral factors (11, 14).

Esketamine, an S-enantiomer of ketamine, functions as a non-competitive NMDA receptor antagonist, inhibiting the action of glutamate and leading to increased levels of glutamate in the synaptic cleft (15, 16). The blockade of NMDA receptors by esketamine shifts signaling to AMPA receptors, enhancing 5HT1B receptor activity, which is hypothesized to be required for its antidepressant effects (16). The activation of AMPA receptors also results in propagation of synaptic plasticity and connectivity, which compounds its antidepressant effects (15, 16).

Esketamine’s ability to produce rapid and sustained antidepressant effects is also linked to its impact on neuroplasticity and neurogenesis. By increasing glutamate cycling in the medial prefrontal cortex and augmenting synaptogenesis and dendritic spine density, esketamine promotes synaptic connectivity and neurogenesis, mediated by brain-derived neurotrophic factor (BDNF) signaling (17). These changes in synaptic plasticity and neurogenesis may also play a role in modulating the neurological and psychological pathways involved in PNES.

**Figure 2:**
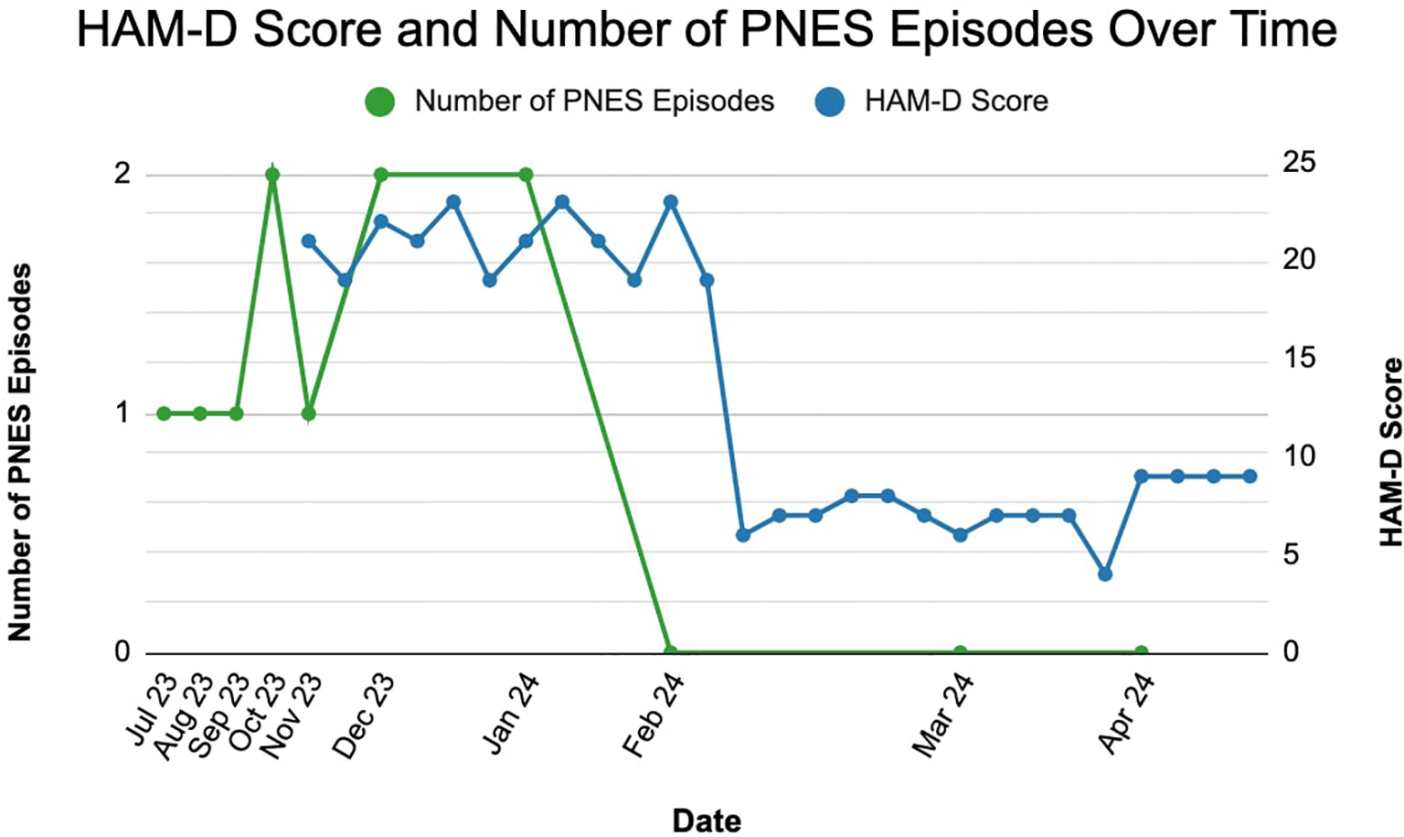
Decrease in hamilton depression rating scale (HAM-D) score and resolution of psychogenic non-epileptic seizures (PNES) following esketamine treatment.

The rapid and robust antidepressant effects of esketamine are well-documented. Studies show that esketamine can reduce depressive symptoms and suicidal ideation within hours of administration, with effects lasting up to a week (17, 18). The high response rates and rapid onset of action make esketamine a valuable option for patients with TRD who have not responded well to other therapies (15). In this case, the patient exhibited significant improvement in depressive symptoms following esketamine treatment, as evidenced by a decrease in her Hamilton Depression Rating Scale (HAM-D) score from 23 pre-treatment to 7 post-treatment, shown in **Figure 2** below. Notably, the patient also reported a resolution of her PNES, which had previously been refractory to multiple antiepileptic and antidepressant medications (**Figure 2**).

The resolution of PNES in this patient following esketamine treatment raises the question of whether esketamine may have therapeutic effects on PNES beyond its antidepressant properties. A possible explanation is that the reduction in depressive symptoms and the alleviation of psychological distress contributed to the resolution of PNES. Given that psychological distress and trauma are significant factors in the etiology of PNES, the antidepressant and anxiolytic effects of esketamine may indirectly reduce the frequency and severity of PNES episodes. This also highlights esketamine’s potential to treat symptoms of PTSD and psychotrauma, as demonstrated in a study reporting significant improvements in both depressive and PTSD symptoms among patients with comorbid TRD and PTSD following esketamine treatment (19). Such effects may be related to ketamine-induced fear extinction, which can help reduce avoidance behaviors in PTSD patients (19).

Moreover, the impact of esketamine on synaptic plasticity and neurogenesis may play a role in modulating the brain regions involved in emotion regulation and stress response, which are critical in the genesis of PNES. Studies suggest that functional changes in the prefrontal cortex, motor regions, thalamic subcortical regions, and hippocampus are implicated in PNES (13, 20). Recent functional magnetic resonance imaging (fMRI) studies on functional neurological disorder (FND), a neuropsychiatric condition of which PNES is a subtype, confirm this (7). Specifically, FND patients were found to exhibit altered co-activation patterns between the insular cortex and the brain’s somatosensory and default mode networks (7). By enhancing synaptic connectivity and neurogenesis in these regions, esketamine may help normalize the brain networks involved in PNES.

There are a number of limitations to this case report. It is based on a single patient, which limits the generalizability of the findings. Larger, more comprehensive studies are needed to validate these observations. Additionally, the findings are not supported by randomized controlled trials (RCTs) and are thus vulnerable to confounding variables. The report also lacks detailed mechanistic insights into how esketamine might affect PNES; understanding the underlying mechanisms would require more in-depth biological, neurological, and psychological studies, including biopsychosocial assessments. Furthermore, the robustness of the observed clinical improvement is limited by the absence of detailed documentation of the patient’s PNES episodes and independent verification through continuous monitoring. Despite these limitations, the information in this report remains valuable in providing preliminary insights and potentially guiding future research into PNES treatment.

## Conclusions

Our report highlights the successful management of PNES and TRD in a 45-year-old female patient using intranasal esketamine. It underscores the potential value of esketamine as a rapid and effective treatment option for TRD with comorbid PNES. This information may be valuable to mental health professionals seeking new therapeutic approaches. However, further research is needed to establish a causal relationship between esketamine treatment and the resolution of PNES, and to explore the underlying mechanisms by which esketamine may influence PNES.

## Data Availability

The original contributions presented in the study are included in the article/supplementary material, further inquiries can be directed to the corresponding author/s.
